# Rapid Spread of Recombinant African Swine Fever Virus Genotypes I and II, Vietnam, 2023–2024

**DOI:** 10.3201/eid3204.251688

**Published:** 2026-04

**Authors:** The Viet Hoang Nguyen, Yeon-Hee Kim, Viet Dung Nguyen, Thi Chau Giang Tran, Ngoc Duong Vu, Thi Thu Hang Vu, Van Tam Nguyen, Da-Young Kim, Ki-Hyun Cho, Seong-Keun Hong, Aruna Ambagala, Van Phan Le

**Affiliations:** Vietnam National University of Agriculture College of Veterinary Medicine, Hanoi, Vietnam (T.V.H Nguyen, V.D. Nguyen, T.C.G. Tran, N.D. Vu, V.P. Le); Animal and Plant Quarantine Agency Foreign Animal Disease Division, Gimcheon, South Korea (Y.-H. Kim, D.-Y. Kim, K.-H. Cho, S.-K. Hong); Vietnam Union of Science and Technology Associations Institute of Veterinary Science and Technology, Hanoi (T.T.H. Vu, V.T. Nguyen); Canadian Food Inspection Agency National Centre for Foreign Animal Disease, Winnipeg, Manitoba, Canada (A. Ambagala)

**Keywords:** African swine fever, viruses, recombinant, rASFV I/II, genetic diversity, genotype, Vietnam

## Abstract

Molecular analyses of African swine fever (ASF) outbreaks in northern and central Vietnam during 2023–2024 revealed a rapid expansion (14.1%–42.2%) of recombinant ASF virus genotypes I and II. Increased prevalence and resistance to commercial ASF vaccines underscore the urgent need for better ASF control and an updated vaccine in Vietnam.

African swine fever (ASF) is a highly contagious viral disease affecting domestic and wild pigs; mortality rates reach up to 100% (*1*). The causative agent, ASF virus (ASFV), is a large double-stranded DNA virus in the *Asfarviridae* family. ASFV strains have been classified into 24 genotypes, of which only genotypes I and II have spread beyond Africa (*2*).

In Asia, ASFV genotype II was first detected in China in 2018 (*3*) and in Vietnam in early 2019 (*4*). Since 2021, recombinant ASFV strains containing genetic elements from genotypes I and II (rASFV I/II) have emerged in China and subsequently in Vietnam in 2023, presenting new challenges for ASF control (*5*,*6*). Two live attenuated ASFV genotype II vaccines have been licensed in Vietnam; however, they provide protection only against homologous strains (*7*,*8*) and are ineffective against rASFV I/II (*9*). Studies conducted during 2019–2022 investigated the molecular epidemiology of ASFV in Vietnam (*10*,*11*), but little is known about circulating strains during 2023–2024. In our study, we aimed to characterize ASFV strains causing outbreaks in northern and central Vietnam during 2023–2024, focusing on the emergence and spread of rASFV I/II.

## The Study

During January 2023–August 2024, we collected 182 clinical samples (spleen, lymph nodes, lung, and whole blood) from pigs suspected of having ASFV infection across 26 provinces in northern and central Vietnam (Appendix Figure 1). We extracted total nucleic acids and tested them by using real-time PCR, targeting the B646L (p72) gene (VDx ASFV qPCR version 2.1; Median Diagnostics, http://mediandiagnostics.com). All samples tested positive for ASFV DNA; cycle threshold values ranged from 14.96 to 32 (Appendix Table). We performed in-house genotyping assays (Phan G1 and G2 Realtime-PCR) to differentiate ASFV genotypes I and II, as previously described (*9*). Results showed that 133/182 samples (73.08%) contained genotype II p72 and 49/182 samples (26.92%) contained genotype I p72. To confirm those findings, we conducted conventional PCRs targeting partial B646L (p72), complete E183L (p54), complete EP402R (CD2v), intergenic region (IGR), and central variable region (CVR), as previously described (*11*). We performed Sanger sequencing on amplicons (1st BASE, https://base-asia.com) and analyzed them by using Geneious Prime 2024 (Geneious, https://www.geneious.com), GraphPad Prism 8.4.3 (GraphPad Software, https://www.graphpad.com), and MEGA 11 (MEGA Software, https://www.megasoftware.net). We visualized phylogenetic trees by using iTOL version 7.4 (Interactive Tree of Life, https://itol.embl.de). We submitted all sequences to GenBank (accession nos. PV975276–639 and PX205390–33).

**Figure 1:**
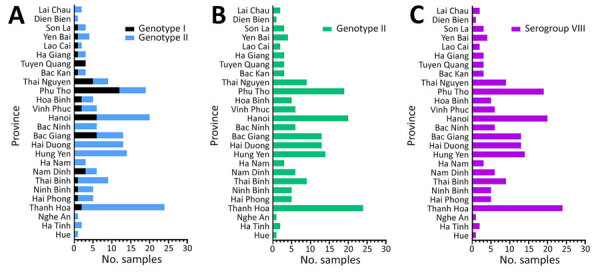
Prevalence and distribution of African swine fever virus genotypes I and II among strains detected, by province, Vietnam, January 2023–August 2024. Genotypes were based on the p72 gene (A) and p54 gene (B) and serogroup classification was based on the CD2v gene (C).

Sequence analysis confirmed that Phan G2–positive samples contained the p72 gene of ASFV genotype II, whereas Phan G1–positive samples carried the p72 gene of genotype I. Of note, all genotype I p72–positive samples also possessed the p54 gene of genotype II and the CD2v gene of serogroup VIII (Figure 1; Appendix Figure 2), consistent with previously reported rASFV I/II strains (*5*,*6*). We detected rASFV I/II in 17 of 26 provinces, whereas genotype II was present nationwide. We identified rASFV I/II in Bac Giang Province in May 2023, four months earlier than previously reported (*5*). A recent study demonstrated high levels of recombination between closely related ASFV genotypes in vitro and in vivo (*12*). In our study, the co-circulation of rASFV I/II and genotype II across multiple provinces suggests an increased risk for further recombination arising from co-infection, particularly in high-density production systems characterized by repeated exposure and prolonged environmental virus persistence. We further characterized ASFV strains on the basis of tandem repeat sequences in the IGR and amino acid variations in the CVR of the B602L gene. To date, 4 IGR variants (IGR I–IV) have been described, and in Vietnam, all 4 IGR types have been reported (*10*). In our study, among the 133 genotype II viruses analyzed, we identified 3 IGR variants (IGR I–III) that showed no intravariant nucleotide differences. IGR II remained dominant, being detected in all 26 provinces, whereas IGR III appeared in only 2 samples (from Bac Giang and Ha Nam Provinces). We found IGR I in 18 samples from 8 provinces (Appendix Figure 3). Those findings suggest a shift in IGR variant distribution among genotype II strains in Vietnam. In contrast, all rASFV I/II strains carried only the IGR II variant, consistent with previous reports (*5*).

Analysis of the B602L CVR region revealed 2 variants (CVR14 and CVR15) among genotype II viruses and 13 variants among rASFV I/II strains (Appendix Figure 4). Genotype II viruses contained 7–10 tetrameric amino acid repeats, whereas rASFV I/II exhibited 42–70 repeats, reflecting greater genetic diversity. The CVR14 variant dominated genotype II strains (131/133), whereas CVR15 was rare. Among rASFV I/II, CVR4 and CVR11 were the most prevalent, being detected across several provinces. Provinces such as Tuyen Quang, Thai Nguyen, Phu Tho, Hanoi, Bac Giang, Nam Dinh, and Thanh Hoa harbored multiple CVR variants, indicating ongoing viral diversification. Some CVR variants showed homology with previously reported strains: CVR2 matched the rASFV strain pig/Henan/123014/2022 from China (*6*), CVR4 corresponded to the Vietnam strain VNUA/rASFV/HD1/23 (*5*), and CVR3 resembled genotype I ASFV OURT 88/3 (*13*). Detection of 13 CVR variants among rASFV I/II strains in 2024 indicates ongoing ASFV evolution and underscores the need to clarify the relationship between genetic diversity and virulence among ASFV strains currently circulating in Vietnam.

**Figure 2:**
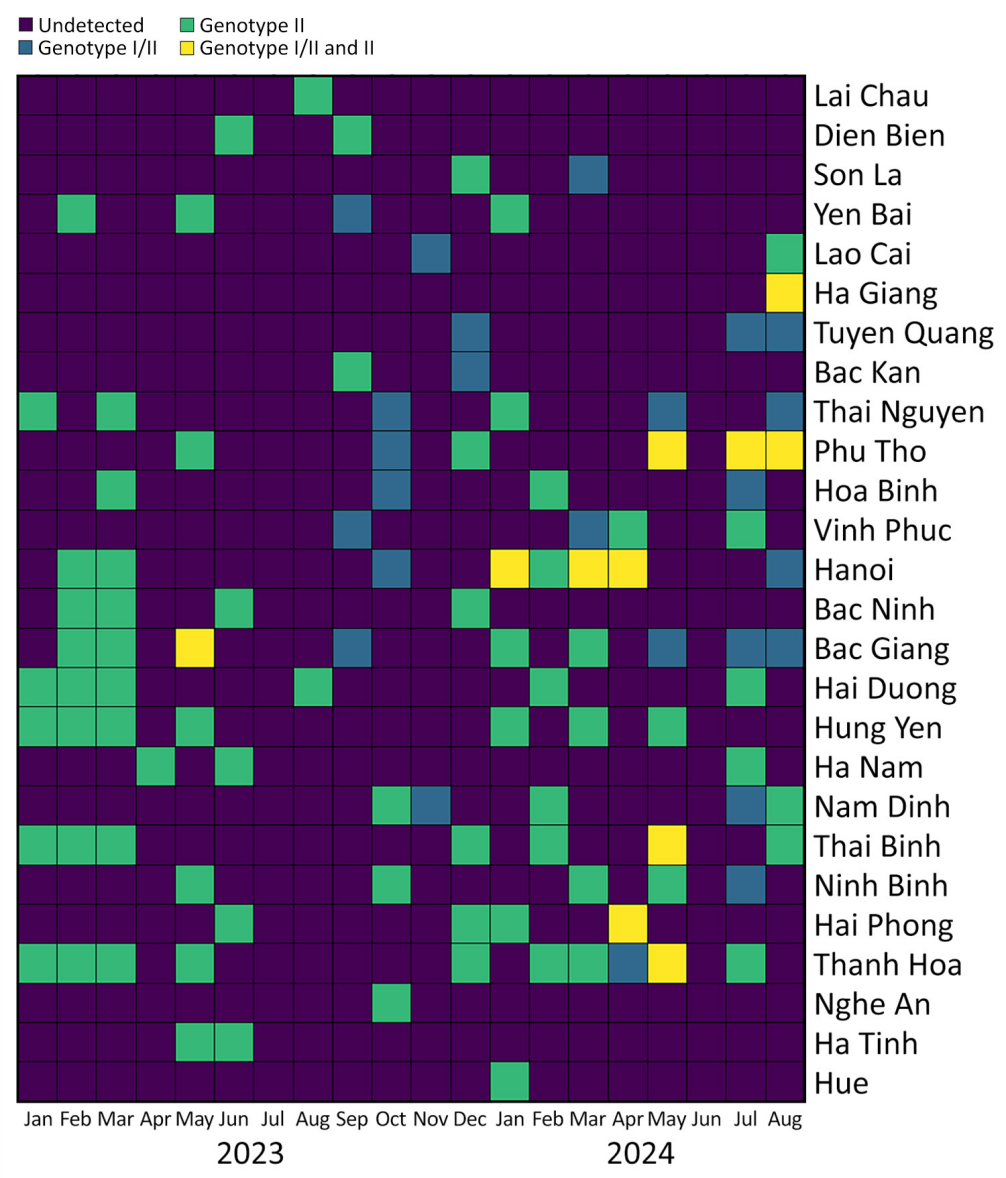
Spatial and temporal distribution of African swine fever virus genotype II and recombinant African swine fever virus strains detected, by province, Vietnam, January 2023–August 2024.

Spatial and temporal analyses showed a statistically significant increase (χ^2^ 17.7; p<0.001) in rASFV I/II among ASFV-positive samples submitted for laboratory diagnosis and confirmation (Figure 2). We detected rASFV I/II in 17 of 26 provinces and observed the proportion increasing from 14.14% (14/99 samples) in 2023 to 42.17% (35/83 samples) in 2024 (Table). That trend suggests rapid geographic expansion and a potential replacement of ASFV genotype II by rASFV I/II. However, those results should be interpreted with caution because they are based on laboratory submissions rather than systematic field surveillance. The mechanisms underlying the shift are unclear but could be attributable to resistance to the vaccines, higher virulence, increased viral shedding, or greater transmission efficiency of the rASFV I/II strains.

**Table:** Circulation rates of African swine fever virus p72 genotype II and recombinant p72 genotype I and II strains detected, Vietnam, January 2023–August 2024

Period	No. samples collected	Genotypes	No. positive	Positivity rate, %
Jan–Dec 2023	99	Genotype I and II	14	14.14
		Genotype II	85	85.86
Jan–Aug 2024	83	Genotype I and II	35	42.17
		Genotype II	48	57.83
Total	182			

## Conclusions

Our study provides strong evidence that highly pathogenic rASFV I/II strains are rapidly evolving and spreading in northern and central Vietnam, showing indications of partial displacement of classical genotype II viruses. Detection of rASFV I/II as early as May 2023 suggests these recombinant strains circulated months before official reporting, highlighting the need for continuous genetic surveillance for early detection. The widespread co-circulation of genotype II and rASFV I/II across multiple provinces underscores the importance of genotyping-based real-time PCR in routine monitoring. Limited cross-protection of existing vaccines emphasizes the urgency of updated immunization strategies and novel vaccine platforms targeting rASFV I/II. Moreover, marked genetic heterogeneity, particularly in CVR regions, indicates ongoing viral evolution with potential effects on virulence and transmission. The co-circulation of multiple ASFV strains raises concern over the emergence of new recombinant variants, complicating disease control. Enhanced genomic surveillance, improved diagnostic tools, and systematic vaccine evaluation are therefore essential for effective ASF prevention and control in Vietnam and the region.

Appendix Additional information about rapid spread of recombinant african swine fever virus genotypes I and II, Vietnam, 2023–2024.
