# Supplementary material for: Rapid Spread of Recombinant African Swine Fever Virus Genotypes I and II, Vietnam, 2023–2024
Source: Emerg Infect Dis. 2026 Apr;32(4):649–52. doi: 10.3201/eid3204.251688 (PMC13094834; doi:10.3201/eid3204.251688)
Supplement: Appendix — Additional information about rapid spread of recombinant african swine fever virus genotypes I and II, Vietnam, 2023–2024. [file 25-1688-Techapp-s1.pdf]

Article DOI: https://doi.org/10.3201/eid3204.251688

EID cannot ensure accessibility for supplementary materials supplied by authors. Readers who have difficulty accessing supplementary content should contact the authors for assistance.

Rapid Spread of Recombinant African Swine Fever Virus Genotypes I and II, Vietnam, 2023–2024

Appendix

Appendix Table. Characteristics of samples included in the study, Vietnam, January 2023–August 2024

						p72 (B646L gen)		p54 (E183L gen)		CD2v (EP402R gen)		CVR (pB602L gen)		IGR I73R/I329L	
No.	Isolate name	Type of sample	Province	Date of collection (d/m/y)	Cq value	Genotype	Acc No.	Genotype	Acc No.	Genotype	Acc No.	Genotype	Acc No.	Genotype	Acc No.
1	VNUA/ASFV/HD1/23	Whole blood	Hai Duong	03 January 2023	22	II	PX205472	II	PV975279	VIII	PV975608	CVR14	PX205885	I	PX205751
2	VNUA/ASFV/HY1/23	Whole blood	Hung Yen	03 January 2023	20.3	II	PX205446	II	PV975288	VIII	PV975519	CVR14	PX205852	II	PX205700
3	VNUA/ASFV/TB1/23	Lympho	Thai Binh	04 January 2023	16.2	II	PX205545	II	PV975400	VIII	PV975522	CVR14	PX205783	II	PX205655
4	VNUA/ASFV/TN1/23	Whole blood	Thai Nguyen	05 January 2023	22.7	II	PX205481	II	PV975405	VIII	PV975624	CVR14	PX205837	II	PX205585
5	VNUA/ASFV/TH1/23	Whole blood	Thanh Hoa	10 January 2023	25.6	II	PX205432	II	PV975369	VIII	PV975577	CVR14	PX205772	II	PX205640
6	VNUA/ASFV/TH2/23	Whole blood	Thanh Hoa	10 January 2023	27.66	II	PX205505	II	PV975334	VIII	PV975571	CVR14	PX205825	II	PX205680
7	VNUA/ASFV/HD2/23	Whole blood	Hai Duong	13 January 2023	27.1	II	PX205393	II	PV975455	VIII	PV975475	CVR14	PX205873	I	PX205743
8	VNUA/ASFV/HD3/23	Whole blood	Hai Duong	13 January 2023	17.8	II	PX205405	II	PV975363	VIII	PV975632	CVR14	PX205784	I	PX205739
9	VNUA/ASFV/HD4/23	Spleen	Hai Duong	16 January 2023	17.3	II	PX205450	II	PV975403	VIII	PV975480	CVR14	PX205790	II	PX205672
10	VNUA/ASFV/HD5/23	Spleen	Hai Duong	17 January 2023	19.6	II	PX205529	II	PV975332	VIII	PV975619	CVR14	PX205819	II	PX205595
11	VNUA/ASFV/HY2/23	Lympho	Hung Yen	30 January 2023	19.8	II	PX205517	II	PV975293	VIII	PV975623	CVR14	PX205804	I	PX205749
12	VNUA/ASFV/HD6/23	Whole blood	Hai Duong	30 January 2023	24.4	II	PX205515	II	PV975401	VIII	PV975479	CVR14	PX205799	I	PX205745
13	VNUA/ASFV/HY3/23	Lung	Hung Yen	02 February 2023	18	II	PX205480	II	PV975410	VIII	PV975530	CVR14	PX205791	II	PX205726
14	VNUA/ASFV/HY4/23	Whole blood	Hung Yen	02 February 2023	31.6	II	PX205564	II	PV975314	VIII	PV975488	CVR14	PX205864	II	PX205651
15	VNUA/ASFV/HN1/23	Spleen	Hanoi	03 February 2023	18.4	II	PX205563	II	PV975364	VIII	PV975570	CVR14	PX205883	II	PX205654
16	VNUA/ASFV/YB1/23	Whole blood	Yen Bai	06 February 2023	27.6	II	PX205419	II	PV975329	VIII	PV975518	CVR14	PX205808	I	PX205742
17	VNUA/ASFV/BG1/23	Lung	Bac Giang	07 February 2023	20.7	II	PX205412	II	PV975349	VIII	PV975508	CVR14	PX205865	II	PX205676
18	VNUA/ASFV/HN2/23	Whole blood	Hanoi	09 February 2023	20.8	II	PX205440	II	PV975386	VIII	PV975597	CVR14	PX205776	II	PX205657
19	VNUA/ASFV/TH3/23	Lympho	Thanh Hoa	10 February 2023	19.3	II	PX205485	II	PV975291	VIII	PV975503	CVR14	PX205764	II	PX205612
20	VNUA/ASFV/TB2/23	Whole blood	Thai Binh	13 February 2023	23.2	II	PX205395	II	PV975336	VIII	PV975462	CVR14	PX205826	II	PX205626
21	VNUA/ASFV/TH4/23	Whole blood	Thanh Hoa	15 February 2023	27.4	II	PX205492	II	PV975352	VIII	PV975600	CVR14	PX205850	II	PX205619
22	VNUA/ASFV/TH5/23	Whole blood	Thanh Hoa	15 February 2023	27	II	PX205402	II	PV975431	VIII	PV975514	CVR14	PX205815	II	PX205584
23	VNUA/ASFV/TH6/23	Lympho	Thanh Hoa	15 February 2023	19.2	II	PX205568	II	PV975289	VIII	PV975473	CVR14	PX205829	II	PX205579
24	VNUA/ASFV/BN1/23	Lympho	Bac Ninh	18 February 2023	17.7	II	PX205437	II	PV975319	VIII	PV975533	CVR14	PX205855	II	PX205699
25	VNUA/ASFV/BN2/23	Whole blood	Bac Ninh	18 February 2023	28.16	II	PX205525	II	PV975354	VIII	PV975569	CVR14	PX205806	II	PX205576
26	VNUA/ASFV/HD7/23	Lung	Hai Duong	19 February 2023	19	II	PX205463	II	PV975310	VIII	PV975537	CVR14	PX205840	I	PX205752
27	VNUA/ASFV/BN3/23	Whole blood	Bac Ninh	20 February 2023	29.6	II	PX205534	II	PV975452	VIII	PV975474	CVR14	PX205777	II	PX205718
28	VNUA/ASFV/TH7/23	Whole blood	Thanh Hoa	22 February 2023	19	II	PX205538	II	PV975306	VIII	PV975512	CVR14	PX205820	II	PX205625
29	VNUA/ASFV/TH8/23	Whole blood	Thanh Hoa	22 February 2023	26.3	II	PX205426	II	PV975424	VIII	PV975472	CVR14	PX205759	II	PX205613
30	VNUA/ASFV/TH9/23	Whole blood	Thanh Hoa	22 February 2023	23.4	II	PX205496	II	PV975295	VIII	PV975592	CVR14	PX205843	II	PX205734

						p72 (B646L gen)		p54 (E183L gen)		CD2v (EP402R gen)		CVR (pB602L gen)		IGR I73R/I329L	
No.	Isolate name	Type of sample	Province	Date of collection (d/m/y)	Cq value	Genotype	Acc No.	Genotype	Acc No.	Genotype	Acc No.	Genotype	Acc No.	Genotype	Acc No.
31	VNUA/ASFV/HY5/23	Spleen	Hung Yen	23 February 2023	19.2	II	PX205433	II	PV975385	VIII	PV975565	CVR14	PX205765	II	PX205589
32	VNUA/ASFV/HY6/23	Spleen	Hung Yen	23 February 2023	19.5	II	PX205401	II	PV975412	VIII	PV975604	CVR14	PX205866	II	PX205641
33	VNUA/ASFV/HN3/23	Whole blood	Hanoi	23 February 2023	18.2	II	PX205410	II	PV975402	VIII	PV975481	CVR14	PX205766	II	PX205711
34	VNUA/ASFV/HN4/23	Whole blood	Hanoi	27 February 2023	26.6	II	PX205473	II	PV975421	VIII	PV975636	CVR15	PX205828	II	PX205643
35	VNUA/ASFV/HD8/23	Whole blood	Hai Duong	27 February 2023	26.6	II	PX205456	II	PV975359	VIII	PV975548	CVR14	PX205816	II	PX205604
36	VNUA/ASFV/HD9/23	Whole blood	Hai Duong	01 March 2023	22.9	II	PX205418	II	PV975394	VIII	PV975555	CVR14	PX205788	II	PX205684
37	VNUA/ASFV/HN5/23	Spleen	Hanoi	05 March 2023	17.8	II	PX205553	II	PV975449	VIII	PV975591	CVR14	PX205844	II	PX205690
38	VNUA/ASFV/HN6/23	Whole blood	Hanoi	08 March 2023	23.4	II	PX205504	II	PV975283	VIII	PV975631	CVR14	PX205851	II	PX205712
39	VNUA/ASFV/BG2/23	Whole blood	Bac Giang	13 March 2023	22	II	PX205497	II	PV975317	VIII	PV975589	CVR14	PX205822	II	PX205609
40	VNUA/ASFV/HY7/23	Whole blood	Hung Yen	14 March 2023	20	II	PX205449	II	PV975299	VIII	PV975491	CVR14	PX205872	II	PX205674
41	VNUA/ASFV/HD10/23	Lung	Hai Duong	14 March 2023	19	II	PX205562	II	PV975392	VIII	PV975602	CVR14	PX205756	II	PX205709
42	VNUA/ASFV/BN4/23	Whole blood	Bac Ninh	14 March 2023	30.21	II	PX205523	II	PV975355	VIII	PV975595	CVR14	PX205876	II	PX205716
43	VNUA/ASFV/TH10/23	Whole blood	Thanh Hoa	14 March 2023	27.7	II	PX205394	II	PV975382	VIII	PV975639	CVR14	PX205860	II	PX205628
44	VNUA/ASFV/TB3/23	Whole blood	Thai Binh	16 March 2023	29	II	PX205511	II	PV975371	VIII	PV975549	CVR14	PX205773	II	PX205685
45	VNUA/ASFV/BG3/23	Spleen	Bac Giang	16 March 2023	17.33	II	PX205396	II	PV975344	VIII	PV975509	CVR14	PX205862	II	PX205720
46	VNUA/ASFV/TN2/23	Whole blood	Thai Nguyen	18 March 2023	32	II	PX205513	II	PV975285	VIII	PV975630	CVR14	PX205853	I	PX205736
47	VNUA/ASFV/TH11/23	Whole blood	Thanh Hoa	18 March 2023	24.9	II	PX205520	II	PV975311	VIII	PV975559	CVR14	PX205833	II	PX205701
48	VNUA/ASFV/TH12/23	Whole blood	Thanh Hoa	18 March 2023	21.2	II	PX205428	II	PV975398	VIII	PV975502	CVR14	PX205814	II	PX205673
49	VNUA/ASFV/TN3/23	Whole blood	Thai Nguyen	18 March 2023	31.64	II	PX205552	II	PV975389	VIII	PV975562	CVR14	PX205770	I	PX205744
50	VNUA/ASFV/TH13/23	Whole blood	Thanh Hoa	20 March 2023	17.96	II	PX205413	II	PV975302	VIII	PV975470	CVR14	PX205886	II	PX205572
51	VNUA/ASFV/TH14/23	Whole blood	Thanh Hoa	21 March 2023	22.6	II	PX205397	II	PV975396	VIII	PV975540	CVR14	PX205760	II	PX205632
52	VNUA/ASFV/HB1/23	Spleen	Hoa Binh	23 March 2023	18	II	PX205571	II	PV975375	VIII	PV975551	CVR14	PX205827	II	PX205653
53	VNUA/ASFV/TH15/23	Whole blood	Thanh Hoa	26 March 2023	25.01	II	PX205559	II	PV975454	VIII	PV975553	CVR14	PX205811	II	PX205659
54	VNUA/ASFV/TH16/23	Lympho	Thanh Hoa	26 March 2023	20.32	II	PX205460	II	PV975324	VIII	PV975515	CVR14	PX205875	II	PX205630
55	VNUA/ASFV/TB4/23	Whole blood	Thai Binh	27 March 2023	19	II	PX205503	II	PV975380	VIII	PV975538	CVR14	PX205782	II	PX205714
56	VNUA/ASFV/HB2/23	Lympho	Hoa Binh	29 March 2023	19	II	PX205441	II	PV975328	VIII	PV975576	CVR14	PX205849	II	PX205600
57	VNUA/ASFV/BG4/23	Whole blood	Bac Giang	30 March 2023	23	II	PX205478	II	PV975450	VIII	PV975609	CVR14	PX205845	II	PX205689
58	VNUA/ASFV/HNa1/23	Lympho	Ha Nam	06 April 2023	22,3	II	PX205407	II	PV975413	VIII	PV975564	CVR14	PX205818	II	PX205591
59	VNUA/ASFV/NB1/23	Spleen	Ninh Binh	03 May 2023	18,6	II	PX205422	II	PV975456	VIII	PV975603	CVR14	PX205839	I	PX205750
60	VNUA/ASFV/HT1/23	Lympho	Ha Tinh	08 May 2023	18,6	II	PX205469	II	PV975280	VIII	PV975614	CVR14	PX205856	II	PX205608
61	VNUA/ASFV/YB2/23	Whole blood	Yen Bai	10 May 2023	28,2	II	PX205498	II	PV975448	VIII	PV975561	CVR14	PX205785	II	PX205681
62	VNUA/ASFV/BG5/23	Whole blood	Bac Giang	16 May 2023	21,01	I	PX205398	II	PV975457	VIII	PV975490	CVR4	PX205920	II	PX205687
63	VNUA/ASFV/TH17/23	Spleen	Thanh Hoa	19 May 2023	17.3	II	PX205406	II	PV975292	VIII	PV975500	CVR14	PX205836	II	PX205682
64	VNUA/ASFV/HY8/23	Whole blood	Hung Yen	20 May 2023	17,9	II	PX205516	II	PV975331	VIII	PV975628	CVR14	PX205813	II	PX205582
65	VNUA/ASFV/PT1/23	Lung	Phu Tho	22 May 2023	19,5	II	PX205528	II	PV975377	VIII	PV975458	CVR14	PX205755	II	PX205647
66	VNUA/ASFV/HNa2/23	Lung	Ha Nam	23 June 2023	18,9	II	PX205403	II	PV975426	VIII	PV975469	CVR14	PX205781	II	PX205679
67	VNUA/ASFV/BN5/23	Whole blood	Bac Ninh	29 June 2023	25,2	II	PX205521	II	PV975381	VIII	PV975476	CVR14	PX205768	II	PX205670
68	VNUA/ASFV/HT2/23	Whole blood	Ha Tinh	03 July 2023	24	II	PX205447	II	PV975316	VIII	PV975557	CVR14	PX205801	II	PX205637
69	VNUA/ASFV/DB1/23	Whole blood	Dien Bien	12 July 2023	23	II	PX205452	II	PV975441	VIII	PV975633	CVR14	PX205763	II	PX205732
70	VNUA/ASFV/HP1/23	Spleen	Hai Phong	25 July 2023	17.3	II	PX205542	II	PV975411	VIII	PV975521	CVR14	PX205861	I	PX205741
71	VNUA/ASFV/LC1/23	Whole blood	Lai Chau	23 August 2023	17.8	II	PX205471	II	PV975340	VIII	PV975535	CVR14	PX205794	II	PX205601
72	VNUA/ASFV/LC2/23	Spleen	Lai Chau	24 August 2023	17.2	II	PX205483	II	PV975438	VIII	PV975635	CVR14	PX205800	II	PX205631
73	VNUA/ASFV/HD11/23	Lympho	Hai Duong	25 August 2023	23.9	II	PX205420	II	PV975407	VIII	PV975566	CVR14	PX205877	II	PX205668
74	VNUA/ASFV/YB4/23	Whole blood	Yen Bai	06 September 2023	18.3	I	PX205461	II	PV975433	VIII	PV975516	N/A	N/A	II	PX205590
75	VNUA/ASFV/HG1/23	Whole blood	Ha Giang	10 September 2023	24.9	II	PX205444	II	PV975374	VIII	PV975563	CVR14	PX205871	II	PX205667
76	VNUA/ASFV/BK1/23	Whole blood	Bac Kan	10 September 2023	17,11	II	PX205429	II	PV975422	VIII	PV975552	CVR14	PX205807	II	PX205707
77	VNUA/ASFV/BG8/23	Spleen	Bac Giang	16 September 2023	17.9	I	PX205514	II	PV975351	VIII	PV975477	CVR4	PX205904	II	PX205624
78	VNUA/ASFV/BG6/23	Lympho	Bac Giang	19 September 2023	18,61	I	PX205391	II	PV975322	VIII	PV975541	CVR4	PX205909	II	PX205581

No.	Isolate name	Type of sample	Province	Date of collection (d/m/y)	Cq value	p72 (B646L gen)		p54 (E183L gen)		CD2v (EP402R gen)		CVR (pB602L gen)		IGR I73R/I329L	
						Genotype	Acc No.	Genotype	Acc No.	Genotype	Acc No.	Genotype	Acc No.	Genotype	Acc No.
79	VNUA/ASFV/VP1/23	Whole blood	Vinh Phuc	20 September 2023	24.7	I	PX205434	II	PV975362	VIII	PV975459	CVR4	PX205899	II	PX205677
80	VNUA/ASFV/BK2/23	Whole blood	Bac Kan	29 September 2023	19.32	II	PX205561	II	PV975333	VIII	PV975485	CVR14	PX205812	II	PX205665
81	VNUA/ASFV/TN4/23	Whole blood	Thai Nguyen	05 October 2023	20,61	I	PX205409	II	PV975436	VIII	PV975627	CVR2	PX205925	II	PX205588
82	VNUA/ASFV/PT3/23	Lympho	Phu Tho	10 October 2023	17,6	I	PX205548	II	PV975341	VIII	PV975590	CVR4	PX205918	II	PX205722
83	VNUA/ASFV/HN7/23	Whole blood	Hanoi	11 October 2023	22,57	I	PX205457	II	PV975330	VIII	PV975621	CVR4	PX205928	II	PX205610
84	VNUA/ASFV/PT4/23	Spleen	Phu Tho	12 October 2023	17,9	I	PX205487	II	PV975325	VIII	PV975494	N/A	N/A	II	PX205678
85	VNUA/ASFV/ND1/23	Whole blood	Nam Dinh	13 October 2023	19.82	II	PX205448	II	PV975390	VIII	PV975598	CVR14	PX205787	II	PX205573
86	VNUA/ASFV/NA1/23	Spleen	Nghe An	23 October 2023	16,53	II	PX205430	II	PV975429	VIII	PV975601	CVR14	PX205858	II	PX205671
87	VNUA/ASFV/HB3/23	Whole blood	Hoa Binh	30 October 2023	22.49	I	PX205500	II	PV975418	VIII	PV975612	CVR4	PX205907	II	PX205597
88	VNUA/ASFV/NB2/23	Lung	Ninh Binh	30 October 2023	16,86	II	PX205495	II	PV975356	VIII	PV975471	CVR14	PX205792	II	PX205639
89	VNUA/ASFV/LCai1/23	Whole blood	Lao Cai	01 November 2023	21.21	I	PX205501	II	PV975327	VIII	PV975611	CVR4	PX205901	II	PX205636
90	VNUA/ASFV/ND2/23	Whole blood	Nam Dinh	30 November 2023	22,31	I	PX205431	II	PV975373	VIII	PV975544	CVR4	PX205906	II	PX205580
91	VNUA/ASFV/TQ1/23	Spleen	Tuyen Quang	01 December 2023	17,55	I	PX205470	II	PV975290	VIII	PV975492	CVR10	PX205891	II	PX205607
92	VNUA/ASFV/SL1/23	Lympho	Son La	01 December 2023	17,49	II	PX205453	II	PV975417	VIII	PV975523	CVR14	PX205869	II	PX205644
93	VNUA/ASFV/TB5/23	Whole blood	Thai Binh	06 December 2023	20,17	II	PX205547	II	PV975395	VIII	PV975606	CVR14	PX205789	II	PX205642
94	VNUA/ASFV/HP2/23	Whole blood	Hai Phong	06 December 2023	18,15	II	PX205519	II	PV975348	VIII	PV975578	CVR14	PX205824	I	PX205753
95	VNUA/ASFV/TH18/23	Whole blood	Thanh Hoa	06 December 2023	24,75	II	PX205491	II	PV975367	VIII	PV975558	CVR14	PX205786	II	PX205611
96	VNUA/ASFV/BN6/23	Whole blood	Bac Ninh	08 December 2023	23,86	II	PX205464	II	PV975343	VIII	PV975634	CVR14	PX205874	II	PX205650
97	VNUA/ASFV/PT2/23	Spleen	Phu Tho	11 December 2023	18,84	II	PX205502	II	PV975353	VIII	PV975556	CVR14	PX205834	II	PX205575
98	VNUA/ASFV/BK3/23	Whole blood	Bac Kan	11 December 2023	16,41	I	PX205488	II	PV975425	VIII	PV975463	CVR3	PX205810	II	PX205623
99	VNUA/ASFV/SL2/23	Lympho	Son La	11 December 2023	15.58	II	PX205510	II	PV975416	VIII	PV975520	CVR14	PX205879	II	PX205627
100	VNUA/ASFV/HN8/24	Whole blood	Hanoi	15 January 2024	24,73	I	PX205539	II	PV975414	VIII	PV975588	CVR4	PX205911	II	PX205614
101	VNUA/ASFV/HY9/24	Spleen	Hung Yen	22 January 2024	16,51	II	PX205445	II	PV975383	VIII	PV975554	CVR14	PX205857	II	PX205666
102	VNUA/ASFV/TN5/24	Lympho	Thai Nguyen	24 January 2024	16,57	II	PX205458	II	PV975350	VIII	PV975496	CVR14	PX205841	I	PX205746
103	VNUA/ASFV/HY10/24	Whole blood	Hung Yen	25 January 2024	18.9	II	PX205550	II	PV975437	VIII	PV975594	CVR14	PX205867	II	PX205724
104	VNUA/ASFV/HN9/24	Whole blood	Hanoi	26 January 2024	22.9	II	PX205467	II	PV975444	VIII	PV975467	CVR14	PX205888	I	PX205740
105	VNUA/ASFV/HP3/24	Lympho	Hai Phong	27 January 2024	28.5	II	PX205392	II	PV975294	VIII	PV975506	CVR14	PX205838	I	PX205747
106	VNUA/ASFV/YB3/24	Whole blood	Yen Bai	27 January 2024	24.2	II	PX205489	II	PV975370	VIII	PV975616	CVR14	PX205771	II	PX205577
107	VNUA/ASFV/Hue1/24	Spleen	Hue	29 January 2024	18	II	PX205518	II	PV975451	VIII	PV975625	CVR14	PX205758	II	PX205638
108	VNUA/ASFV/BG7/24	Lympho	Bac Giang	29 January 2024	17.4	II	PX205524	II	PV975361	VIII	PV975607	CVR14	PX205878	III	PX205616
109	VNUA/ASFV/TH19/24	Spleen	Thanh Hoa	17 February 2024	18.7	II	PX205476	II	PV975372	VIII	PV975617	CVR14	PX205797	I	PX205748
110	VNUA/ASFV/HD12/24	Lympho	Hai Duong	17 February 2024	19.7	II	PX205551	II	PV975347	VIII	PV975497	CVR14	PX205863	I	PX205737
111	VNUA/ASFV/HB4/24	Whole blood	Hoa Binh	18 February 2024	22.8	II	PX205414	II	PV975305	VIII	PV975637	CVR14	PX205884	II	PX205598
112	VNUA/ASFV/TB6/24	Spleen	Thai Binh	19 February 2024	17.8	II	PX205408	II	PV975277	VIII	PV975572	CVR14	PX205868	II	PX205731
113	VNUA/ASFV/HN10/24	Whole blood	Hanoi	19 February 2024	20.9	II	PX205493	II	PV975309	VIII	PV975505	CVR14	PX205847	I	PX205738
114	VNUA/ASFV/HN11/24	Lympho	Hanoi	20 February 2024	18.8	II	PX205424	II	PV975357	VIII	PV975545	CVR14	PX205775	II	PX205662
115	VNUA/ASFV/ND3/24	Lympho	Nam Dinh	21 February 2024	16.7	II	PX205544	II	PV975345	VIII	PV975638	CVR14	PX205842	II	PX205697
116	VNUA/ASFV/TH20/24	Lympho	Thanh Hoa	02 March 2024	18.43	II	PX205490	II	PV975303	VIII	PV975542	CVR14	PX205859	II	PX205603
117	VNUA/ASFV/HY11/24	Lympho	Hung Yen	02 March 2024	19.01	II	PX205540	II	PV975326	VIII	PV975583	CVR14	PX205796	II	PX205621
118	VNUA/ASFV/BG9/24	Whole blood	Bac Giang	04 March 2024	24.91	II	PX205557	II	PV975440	VIII	PV975568	CVR15	PX205882	II	PX205648
119	VNUA/ASFV/HN12/24	Whole blood	Hanoi	04 March 2024	24.79	II	PX205475	II	PV975423	VIII	PV975525	CVR14	PX205769	II	PX205596
120	VNUA/ASFV/SL3/24	Lympho	Son La	04 March 2024	16.86	I	PX205537	II	PV975308	VIII	PV975461	CVR12	PX205892	II	PX205578
121	VNUA/ASFV/NB3/24	Whole blood	Ninh Binh	06 March 2024	20.29	II	PX205421	II	PV975409	VIII	PV975495	CVR14	PX205757	II	PX205692
122	VNUA/ASFV/BG10/24	Whole blood	Bac Giang	06 March 2024	26.45	II	PX205442	II	PV975434	VIII	PV975493	CVR14	PX205846	II	PX205730
123	VNUA/ASFV/HN13/24	Whole blood	Hanoi	07 March 2024	21,03	II	PX205506	II	PV975284	VIII	PV975626	CVR14	PX205767	II	PX205721
124	VNUA/ASFV/HN14/24	Whole blood	Hanoi	07 March 2024	20.25	II	PX205522	II	PV975365	VIII	PV975486	CVR14	PX205802	II	PX205727
125	VNUA/ASFV/HN15/24	Whole blood	Hanoi	07 March 2024	18,37	II	PX205556	II	PV975453	VIII	PV975605	CVR14	PX205779	II	PX205695
126	VNUA/ASFV/HY12/24	Whole blood	Hung Yen	09 March 2024	21,64	II	PX205416	II	PV975446	VIII	PV975610	CVR14	PX205832	II	PX205594

						p72 (B646L gen)		p54 (E183L gen)		CD2v (EP402R gen)		CVR (pB602L gen)		IGR I73R/I329L	
No.	Isolate name	Type of sample	Province	Date of collection (d/m/y)	Cq value	Genotype	Acc No.	Genotype	Acc No.	Genotype	Acc No.	Genotype	Acc No.	Genotype	Acc No.
127	VNUA/ASFV/VP2/24	Whole blood	Vinh Phuc	09 March 2024	22.99	I	PX205560	II	PV975282	VIII	PV975465	CVR4	PX205912	II	PX205583
128	VNUA/ASFV/HN16/24	Whole blood	Hanoi	15 March 2024	25.75	I	PX205507	II	PV975435	VIII	PV975587	CVR4	PX205924	II	PX205587
129	VNUA/ASFV/HP4/24	Tissue	Hai Phong	06 April 2024	20.82	II	PX205555	II	PV975391	VIII	PV975550	CVR14	PX205793	II	PX205735
130	VNUA/ASFV/VP3/24	Tissue	Vinh Phuc	06 April 2024	17.59	II	PX205443	II	PV975388	VIII	PV975466	CVR14	PX205854	II	PX205661
131	VNUA/ASFV/HP5/24	Tissue	Hai Phong	09 April 2024	17.96	I	PX205455	II	PV975321	VIII	PV975498	CVR4	PX205902	II	PX205717
132	VNUA/ASFV/TH21/24	Tissue	Thanh Hoa	16 April 2024	20.37	I	PX205558	II	PV975427	VIII	PV975581	CVR3	PX205761	II	PX205622
133	VNUA/ASFV/HN17/24	Whole blood	Hanoi	17 April 2024	20.12	I	PX205435	II	PV975297	VIII	PV975560	CVR10	PX205921	II	PX205629
134	VNUA/ASFV/HN18/24	Serum	Hanoi	24 April 2024	22.99	II	PX205462	II	PV975378	VIII	PV975615	CVR14	PX205778	II	PX205704
135	VNUA/ASFV/HY13/24	Whole blood	Hung Yen	02 May 2024	23.91	II	PX205531	II	PV975366	VIII	PV975543	CVR14	PX205880	II	PX205733
136	VNUA/ASFV/TB7/24	Spleen	Thai Binh	02 May 2024	18.29	II	PX205390	II	PV975443	VIII	PV975599	CVR14	PX205887	II	PX205620
137	VNUA/ASFV/HY14/24	Whole blood	Hung Yen	02 May 2024	22.7	II	PX205565	II	PV975281	VIII	PV975531	CVR14	PX205798	II	PX205615
138	VNUA/ASFV/TH22/24	Spleen	Thanh Hoa	07 May 2024	15,69	I	PX205549	II	PV975445	VIII	PV975528	CVR7	PX205910	II	PX205593
139	VNUA/ASFV/TN6/24	Whole blood	Thai Nguyen	07 May 2024	23,83	I	PX205482	II	PV975312	VIII	PV975629	CVR4	PX205905	II	PX205592
140	VNUA/ASFV/PT5/24	Whole blood	Phu Tho	08 May 2024	19.41	I	PX205533	II	PV975346	VIII	PV975579	CVR1	PX205915	II	PX205729
141	VNUA/ASFV/NB4/24	Spleen	Ninh Binh	08 May 2024	17.51	II	PX205474	II	PV975313	VIII	PV975510	CVR14	PX205762	II	PX205606
142	VNUA/ASFV/TB8/24	Whole blood	Thai Binh	10 May 2024	23,01	I	PX205423	II	PV975406	VIII	PV975613	CVR6	PX205929	II	PX205696
143	VNUA/ASFV/PT6/24	Lympho	Phu Tho	10 May 2024	15.36	II	PX205451	II	PV975276	VIII	PV975573	CVR14	PX205870	II	PX205688
144	VNUA/ASFV/TH23/24	Whole blood	Thanh Hoa	10 May 2024	26.17	II	PX205508	II	PV975408	VIII	PV975482	CVR14	PX205831	II	PX205698
145	VNUA/ASFV/BG11/24	Lympho	Bac Giang	10 May 2024	15.38	I	PX205536	II	PV975447	VIII	PV975524	CVR9	PX205930	II	PX205646
146	VNUA/ASFV/PT7/24	Whole blood	Phu Tho	01 July 2024	26.7	II	PX205436	II	PV975315	VIII	PV975547	CVR14	PX205817	II	PX205691
147	VNUA/ASFV/TH24/24	Whole blood	Thanh Hoa	01 July 2024	24.03	II	PX205465	II	PV975301	VIII	PV975574	CVR14	PX205754	II	PX205683
148	VNUA/ASFV/BG12/24	Whole blood	Bac Giang	03 July 2024	18,5	I	PX205546	II	PV975442	VIII	PV975529	CVR4	PX205923	II	PX205693
149	VNUA/ASFV/TQ2/24	Spleen	Tuyen Quang	03 July 2024	15.56	I	PX205554	II	PV975320	VIII	PV975534	CVR4	PX205908	II	PX205656
150	VNUA/ASFV/ND4/24	Whole blood	Nam Dinh	03 July 2024	16.04	I	PX205466	II	PV975376	VIII	PV975501	CVR11	PX205893	II	PX205633
151	VNUA/ASFV/ND5/24	Whole blood	Nam Dinh	03 July 2024	23.47	I	PX205404	II	PV975300	VIII	PV975532	CVR5	PX205897	II	PX205725
152	VNUA/ASFV/VP4/24	Whole blood	Vinh Phuc	04 July 2024	21.35	II	PX205468	II	PV975296	VIII	PV975484	CVR14	PX205805	II	PX205706
153	VNUA/ASFV/PT8/24	Whole blood	Phu Tho	04 July 2024	20.37	I	PX205399	II	PV975360	VIII	PV975478	CVR8	PX205931	II	PX205723
154	VNUA/ASFV/PT9/24	Whole blood	Phu Tho	04 July 2024	20.8	II	PX205532	II	PV975335	VIII	PV975468	CVR14	PX205823	II	PX205669
155	VNUA/ASFV/VP5/24	Whole blood	Vinh Phuc	05 July 2024	26.09	II	PX205454	II	PV975393	VIII	PV975489	CVR14	PX205881	II	PX205617
156	VNUA/ASFV/PT10/24	Spleen	Phu Tho	06 July 2024	16.49	I	PX205567	II	PV975428	VIII	PV975546	CVR6	PX205896	II	PX205660
157	VNUA/ASFV/PT11/24	Spleen	Phu Tho	09 July 2024	15.67	I	PX205494	II	PV975379	VIII	PV975622	CVR5	PX205932	II	PX205602
158	VNUA/ASFV/PT12/24	Whole blood	Phu Tho	10 July 2024	23.69	II	PX205527	II	PV975298	VIII	PV975618	CVR14	PX205780	II	PX205675
159	VNUA/ASFV/VP6/24	Whole blood	Vinh Phuc	10 July 2024	16.74	II	PX205570	II	PV975318	VIII	PV975575	CVR14	PX205821	II	PX205645
160	VNUA/ASFV/HD13/24	Spleen	Hai Duong	11 July 2024	14.96	II	PX205509	II	PV975387	VIII	PV975464	CVR14	PX205795	II	PX205618
161	VNUA/ASFV/HNa3/24	Whole blood	Ha Nam	13 July 2024	20,44	II	PX205541	II	PV975339	VIII	PV975483	CVR14	PX205809	III	PX205605
162	VNUA/ASFV/NB5/24	Whole blood	Ninh Binh	15 July 2024	17,48	I	PX205535	II	PV975338	VIII	PV975526	CVR4	PX205927	II	PX205652
163	VNUA/ASFV/HB5/24	Whole blood	Hoa Binh	16 July 2024	21.57	I	PX205438	II	PV975286	VIII	PV975585	CVR4	PX205889	II	PX205710
164	VNUA/ASFV/PT13/24	Whole blood	Phu Tho	17 July 2024	18.48	I	PX205566	II	PV975419	VIII	PV975539	CVR13	PX205895	II	PX205663
165	VNUA/ASFV/PT14/24	Whole blood	Phu Tho	18 July 2024	16.95	I	PX205512	II	PV975397	VIII	PV975504	CVR5	PX205922	II	PX205686
166	VNUA/ASFV/PT15/24	Whole blood	Phu Tho	03 August 2024	20.38	I	PX205479	II	PV975415	VIII	PV975487	CVR13	PX205894	II	PX205635
167	VNUA/ASFV/LCai2/24	Whole blood	Lao Cai	04 August 2024	19.88	II	PX205530	II	PV975439	VIII	PV975499	CVR14	PX205803	II	PX205715
168	VNUA/ASFV/TN7/24	Whole blood	Thai Nguyen	06 August 2024	18.21	I	PX205484	II	PV975278	VIII	PV975536	CVR4	PX205898	II	PX205705
169	VNUA/ASFV/ND6/24	Spleen	Nam Dinh	06 August 2024	17.26	II	PX205543	II	PV975432	VIII	PV975582	CVR14	PX205835	II	PX205713
170	VNUA/ASFV/PT16/24	Whole blood	Phu Tho	07 August 2024	26,03	I	PX205459	II	PV975420	VIII	PV975593	CVR4	PX205914	II	PX205694
171	VNUA/ASFV/TB9/24	Spleen	Thai Binh	07 August 2024	16,33	II	PX205415	II	PV975342	VIII	PV975584	CVR14	PX205830	II	PX205586
172	VNUA/ASFV/PT17/24	Whole blood	Phu Tho	08 August 2024	24.32	II	PX205569	II	PV975358	VIII	PV975507	CVR14	PX205774	II	PX205702
173	VNUA/ASFV/TN8/24	Spleen	Thai Nguyen	09 August 2024	15.76	I	PX205427	II	PV975430	VIII	PV975567	CVR10	PX205933	II	PX205649
174	VNUA/ASFV/PT18/24	Whole blood	Phu Tho	10 August 2024	18.93	I	PX205486	II	PV975368	VIII	PV975517	CVR5	PX205917	II	PX205728

No.	Isolate name	Type of sample	Province	Date of collection (d/m/y)	Cq value	p72 (B646L gen)		p54 (E183L gen)		CD2v (EP402R gen)		CVR (pB602L gen)		IGR I73R/I329L	
						Genotype	Acc No.	Genotype	Acc No.	Genotype	Acc No.	Genotype	Acc No.	Genotype	Acc No.
175	VNUA/ASFV/HG2/24	Spleen	Ha Giang	11 August 2024	18.58	II	PX205499	II	PV975384	VIII	PV975580	CVR14	PX205848	II	PX205708
176	VNUA/ASFV/TQ3/24	Whole blood	Tuyen Quang	12 August 2024	18.93	I	PX205400	II	PV975404	VIII	PV975513	CVR5	PX205913	II	PX205634
177	VNUA/ASFV/PT19/24	Lympho	Phu Tho	16 August 2024	18.67	I	PX205526	II	PV975304	VIII	PV975460	CVR5	PX205903	II	PX205719
178	VNUA/ASFV/TN9/24	Whole blood	Thai Nguyen	26 August 2024	19.37	I	PX205411	II	PV975323	VIII	PV975527	CVR4	PX205919	II	PX205658
179	VNUA/ASFV/BG13/24	Whole blood	Bac Giang	26 August 2024	19.24	I	PX205439	II	PV975307	VIII	PV975586	CVR2	PX205916	II	PX205664
180	VNUA/ASFV/HG3/24	Whole blood	Ha Giang	29 August 2024	17.58	I	PX205425	II	PV975287	VIII	PV975511	CVR4	PX205900	II	PX205599
181	VNUA/ASFV/HN19/24	Whole blood	Hanoi	30 August 2024	21.19	I	PX205417	II	PV975399	VIII	PV975596	CVR4	PX205926	II	PX205574
182	VNUA/ASFV/HN20/24	Whole blood	Hanoi	30 August 2024	29.47	I	PX205477	II	PV975337	VIII	PV975620	CVR4	PX205890	II	PX205703

Note: N/A = Sequencing failure due to low sequencing quality.

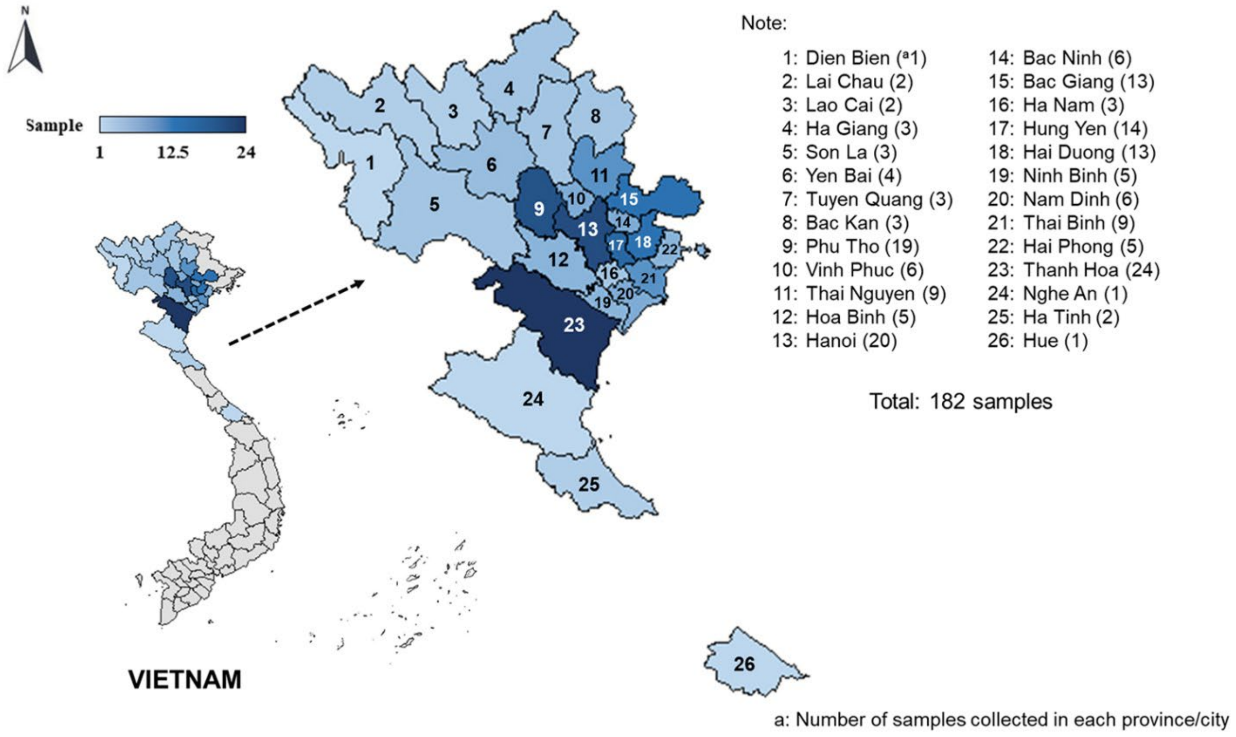

Appendix Figure 1. Map showing the number of samples and locations of ASFV detection in Vietnam from January 2023 to August 2024.

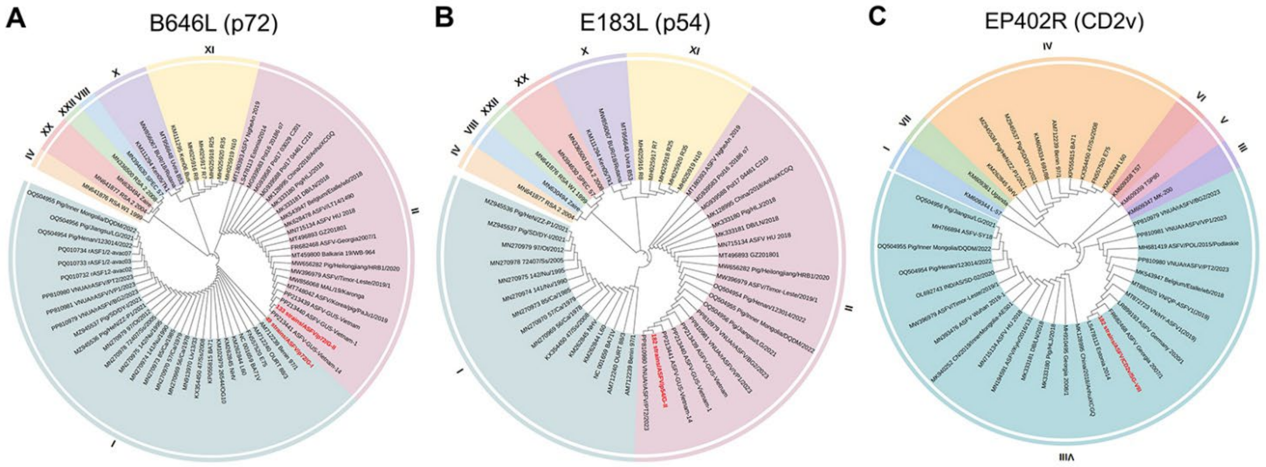

Appendix Figure 2. Phylogenetic trees of African swine fever virus (ASFV) strains detected in Vietnam from January 2023 to August 2024, based on the (A) p72, (B) p54, and (C) CD2v genes. The trees were constructed using the maximum likelihood method with 1,000 bootstrap replicates. ASFV strains from the present study are indicated by red circles.

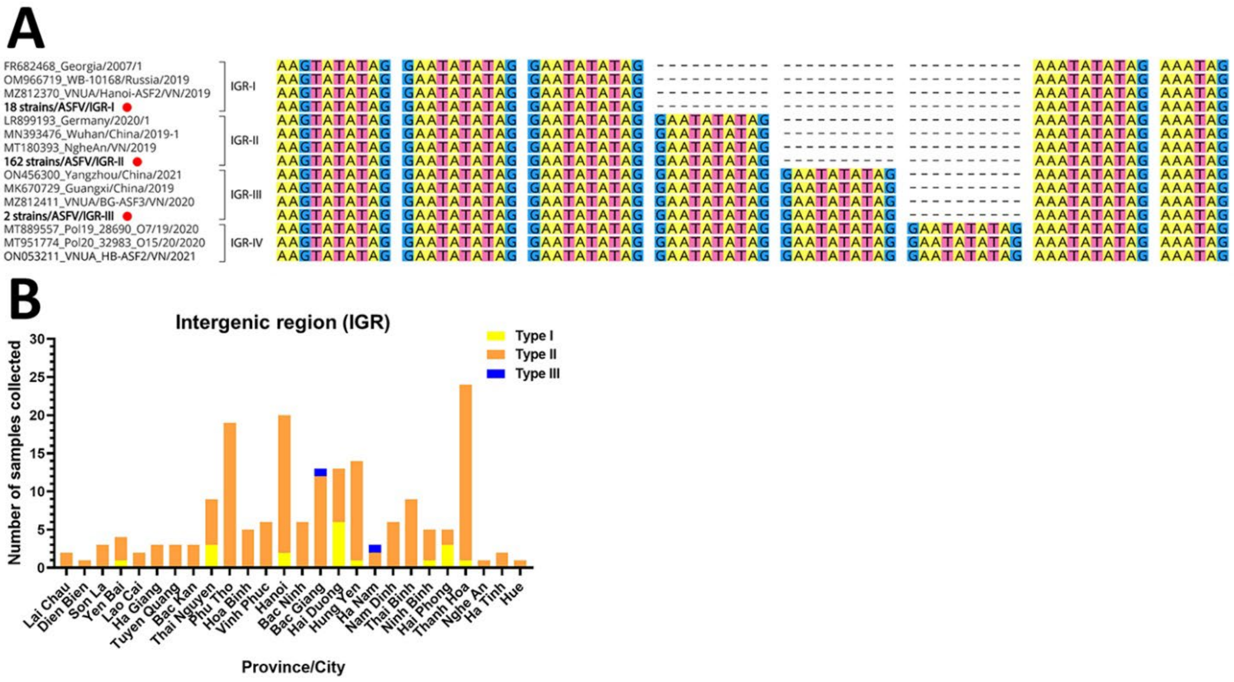

Appendix Figure 3. Sequences and prevalence of the intergenic region (IGR) of ASFV strains from Vietnam from January 2023 to August 2024. (A) Alignment of the IGR sequences showing three distinct variants identified in this study. (B) Geographic distribution of the IGR variants across different locations.

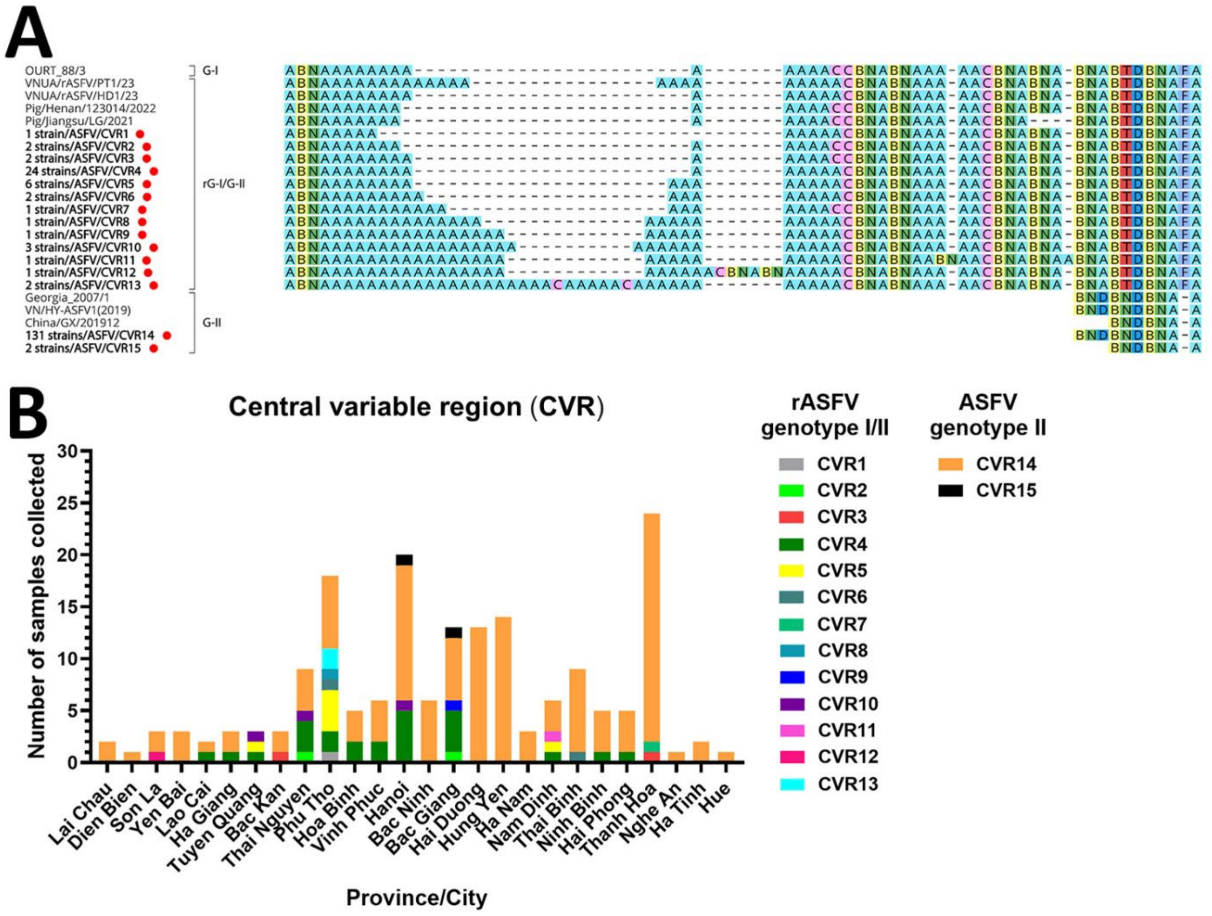

Appendix Figure 4. Sequences and prevalence of the central variable region (CVR) of ASFV isolates from Vietnam from January 2023 to August 2024. (A) Alignment of the CVR showing multiple variants identified in this study. The amino acid sequence alignment of the tetrameric repeats within the central variable region of the B602L gene was performed on 180 ASFV isolates and revealed 15 distinct patterns. The single-letter codes for each tetrameric repeat are as follows: A = CAST/CTST, B = CADT, C = GAST, D = CASM, F = CANT, N = NVDT, and T = NVNT. The study includes ASFV strains from this investigation (highlighted in bold with red dots), previously reported recombinant ASFV strains, and reference strains of genotypes I and II. (B) Geographic distribution of the identified CVR variants.
